# Identification of molecular biomarkers associated with disease progression in the testis of bulls infected with *Besnoitia besnoiti*

**DOI:** 10.1186/s13567-021-00974-2

**Published:** 2021-07-22

**Authors:** David González-Barrio, Carlos Diezma-Díaz, Daniel Gutiérrez-Expósito, Enrique Tabanera, Alejandro Jiménez-Meléndez, Manuel Pizarro, Marta González-Huecas, Ignacio Ferre, Luis M. Ortega-Mora, Gema Álvarez-García

**Affiliations:** 1 SALUVET, Animal Health Department, Faculty of Veterinary Sciences, Complutense University of Madrid, Ciudad Universitaria s/n, 28040 Madrid, Spain; 2 Livestock Health and Production Institute (ULE-CSIC), León, Spain; 3 Department of Animal Medicine and Surgery, Faculty of Veterinary Sciences, Complutense University of Madrid, Ciudad Universitaria s/n, 28040 Madrid, Spain

**Keywords:** *Besnoitia besnoiti*, Bovine besnoitiosis, Breeding bull, Testis, Molecular markers

## Abstract

Breeding bulls infected with *Besnoitia besnoiti* may develop sterility during either acute or chronic infection. The aim of this study was to investigate the molecular pathogenesis of *B. besnoiti* infection with prognosis value in bull sterility. Accordingly, five well-characterized groups of naturally and experimentally infected males were selected for the study based on clinical signs and lesions compatible with *B. besnoiti* infection, serological results and parasite detection. A broad panel of molecular markers representative of endothelial activation and fibrosis was investigated and complemented with a histopathological approach that included conventional histology and immunohistochemistry. The results indicated the predominance of an intense inflammatory infiltrate composed mainly of resident and recruited circulating macrophages and to a lesser extent of CD3+ cells in infected bulls. In addition, a few biomarkers were associated with acute, chronic or subclinical bovine besnoitiosis. The testicular parenchyma showed a higher number of differentially expressed genes in natural infections (acute and chronic infections) versus scrotal skin in experimental infections (subclinical infection). In subclinical infections, most genes were downregulated except for the CCL24 and CXCL2 genes, which were upregulated. In contrast, the acute phase was mainly characterized by the upregulation of IL-1α, IL-6 and TIMP1, whereas in the chronic phase, the upregulation of ICAM and the downregulation of MMP13, PLAT and IL-1α were the most relevant findings. Macrophages could be responsible for the highest level of gene regulation in the testicular parenchyma of severely affected and sterile bulls, and all these genes could be prognostic markers of sterility.

**Supplementary Information:**

The online version contains supplementary material available at 10.1186/s13567-021-00974-2.

## Introduction

Bovine besnoitiosis is caused by the cyst-forming protist coccidian parasite *Besnoitia besnoiti.* It is a well-known endemic cattle disease in sub-Saharan countries [1, 2] and has been spreading throughout different countries in Europe for the last two decades [3, 4] in the absence of vaccines and therapeutic tools. Major concerns have emerged from the disease impact in cattle raised under extensive husbandry systems, as risk factors associated with management measures favour parasite dissemination. Severe economic losses are due to the poor body condition, reduced value of hides for leather production and male sterility, which is a major consequence that impairs the herd fertility rate. In beef cattle herds, males appeared to show acute clinical signs more frequently and higher mortality rates than females, as they might be more exposed to the infection than females due to natural mating [5, 6]. Gazzonis et al. [7] reported that males presented a greater risk of infection, with an incidence of infection of 60% vs 38.8% in females, and a greater risk of spreading the disease to females through natural mating [8].

Clinical besnoitiosis outcome progresses in two sequential phases. The acute stage is caused by the rapid replication and intra-organic dissemination of the tachyzoite stage. It is initially characterized by fever, depression, and anorexia (named the febrile stage) and followed by generalized oedema, ocular and nasal discharge and orchitis, among others (named the anasarca stage) as a consequence of vascular disorders [9]. The chronic stage initiates when most tachyzoites are cleared by the immune response and tachyzoites switch into bradyzoites leading to tissue cyst formation. The acute disease may evolve to the chronic phase in less than one month [10]. Chronic besnoitiosis (scleroderma stage) is characterized by skin lesions such as hyperkeratosis, skin folding and alopecia that occurs as a consequence of the development of tissue cysts in the connective tissues. Pathognomonic tissue cysts are visible in ocular conjunctiva, mucous membranes of the upper respiratory track and *vestibulum vaginae*. Bulls may end up with testicular degeneration and azoospermia.

It was recently reported that bulls may develop sterility during the acute phase, characterized by the coexistence of acute and chronic characteristic lesions (vascular injury in testis tissues and scrotal skin lesions) that hamper testicular thermoregulation. Granulomatous inflammation was also a relevant finding in the testicular parenchyma, but whether it plays a key role on the pathogenesis remains unknown [11]. In chronically infected bulls, characteristic histopathological findings of the chronic disease are predominant. Numerous cysts observed in the testes, epididymis, ampullae and in the walls of blood vessels in the pampiniform plexus may contribute to the thermoregulation failure favoured by intense fibrosis and thickening of the scrotal skin that interfere with normal spermatogenesis [8, 12].

However, the molecular basis governing disease progression and determining severity could help to identify molecular markers of prognosis. Recent in vitro studies proved that *B. besnoiti* infection can modulate endothelial cells. Upon infection, an early activation of endothelial cells is progressively induced with an upregulation of leukocyte adhesion molecules as well as pro-inflammatory and profibrotic phenotypes, which could be responsible for macrophage recruitment [13, 14]. Macrophages are key players in fibrosis pathogenesis, and fibrosis is a typical finding of chronic besnoitiosis. Indeed, angiogenesis and extracellular matrix reorganization were also upregulated pathways in the in vitro bovine endothelial cell model [14]. In this context, a further step is needed in order to elucidate whether these in vitro molecular pathways are found in vivo.

Thus, the aim of this work was to bridge the gap in knowledge about the in vivo molecular pathogenesis of *B. besnoiti* infection, which will have value for predicting the bull sterility. Different testis tissues (scrotal skin, pampiniform plexus and testicular parenchyma) and sera were analysed from males with either natural or experimental *B. besnoiti* infection as well as non-infected males. Males were grouped into the following categories: acutely and chronically infected breeding bulls with impaired fertility and systemic clinical signs and lesions in the testicles; experimentally chronically infected calves with a few relevant microscopic lesions in the testicles; and their respective non-infected control groups from natural and experimental infections. A wide panel of molecular markers representative of endothelial activation, fibrosis, reorganization of the extracellular matrix and cytokines [14, 15] was investigated and complemented with a histopathological approach that included conventional histology and immunohistochemistry. Genes investigated were representative of endothelial activation (leucocyte attraction, a pro-inflammatory response and response against a coagulant state), and fibrosis. These genes were mostly selected based on previous findings of differentially expressed genes in an in vitro model of *B. besnoiti*-infected endothelial cells [14]. Additionally, genes related to the reorganization of the extracellular matrix and regulatory and anti-inflammatory cytokines described in infections by the closely related *Neospora caninum* and *Toxoplasma gondii* parasites were also studied [15, 16].

## Materials and methods

### Males included in the study and samples collected

Five well characterized groups (A-E) of males were selected for the study based on clinical signs and lesions compatible with *B. besnoiti* infection, serological results and parasite detection. A summary of the most relevant pathological findings together with serological and molecular results previously published for groups A, B and C are shown in Table 1. Regarding group D, data on the age, breed, origin, and serological results obtained herein are presented in Table 2.

**Table 1:** **Summary of the most relevant results regarding pathological findings (clinical signs and lesions), serological and molecular results (﻿parasite detection) in experimentally infected groups (A and B), and naturally infected group with an acute infection (group C)**

Study group^1^ (Natural infection -N-/Experimental infection -E-)	Bull (B)/calf (C) number	Infection stage	Clinical signs and lesions		Serological results	Parasite detection	
			Systemic level	Macroscopic/microscopic lesions in the testicles	IgM/IgG/ IgG avidity	PCR^2^	Histopathology^3^
A (E)	C1-3^4^	Subclinical	Congestive ocular sclera, conjunctival cysts	–^5^/Inflammatory infiltrate	+ / + / low avidity	+ / − / −^6^	–
	C4-6		Congestive ocular sclera, conjunctival cysts	–/Inflammatory infiltrate and oedemas	+ / + / low avidity	− / − / −	–
	C7-9		Congestive ocular sclera, conjunctival cysts, skin lesions	–/Skin lesions, inflammatory infiltrate and oedemas	+ / + / low avidity	+ / + / +	131,5 TP; 156,1 SK
B (E)	C1-3	Non-infected	Without clinical signs or lesions	Without lesions	− / − / n.a	−/−/−	–
C (N)	B1	Acute	Fever and edema in posterior limbs	Orchitis/Vascular lesions, inflammatory infiltrate, lesion on scrotal skin, fibroplasia, aspermia	+ / + / low avidity	+ / – / +	50.0 SK
	B2		Respiratory problems, edema in the area of the scapula	nd /Vascular lesions, inflammatory infiltrate, lesion on scrotal skin, fibroplasia, aspermia	No serum available	− / + / +	97.1 PP/20.0 TP/90.7 SK
	B3		Edema in declining areas	Orchitis/Vascular lesions, inflammatory infiltrate	No serum available	+ / + / +	–
	B4		nd	Orchitis and vascular lesions/Vascular lesions, inflammatory infiltrate, lesion on scrotal skin, fibroplasia, aspermia	+ / – /na	+ / + / +	–
	B5		nd	Vascular lesions and adherences in testicles/Vascular lesions, inflammatory infiltrate	+ / – /na	+ / + / +	–
	B6		Fever	Orchitis and vascular lesions/Vascular lesions, inflammatory infiltrate, aspermia	+ / – /na	− / + / +	25.0 SK
	B7		Fever	Orchitis and vascular lesions/Vascular lesions, inflammatory infiltrate, fibroplasia	+ / – /na	− / + / +	–

nd, not determined; na, not applicable.

^1^More detailed data on these selected naturally and experimentally infected animals can be found in González-Barrio et al. [11], and Diezma-Diaz et al. [17, 20, 21].

^2^ + or – mean that pampiniform plexus, testicular parenchyma and scrotal skin are *Besnoitia* spp. PCR positive or negative, respectively.

^3^Average cyst diameter (μm) in SK (scrotal skin), PP (pampiniform plexus), TP (testicular parenchyma); –cysts not found.

^4^Calves C1–3 were inoculated by intravenous route, calves C4-6 were inoculated by subcutaneous route and calves C7-9 were inoculated by intradermal route.

^5^– macroscopic lesions not found.

^6^ + or − mean at least one animal per group was PCR positive or negative, respectively.

**Table 2:** **Chronically infected bulls included in this study**

Bull number	Clinical stage	Age (months)	Breed	Geographical origin	Clinical signs and lesions	Serology					
						IgM ELISA		IgG ELISA		WB	**Avidity**
						RIPC	POS ( +) /NEG (-)	RIPC	POS ( +) /NEG (-)	POS ( +) /NEG (-)	
B8	Chronic	Adult^1^	Charolais	Guadalajara	Folds and hyperkeratosis in scrotum, skin of perineum, carpus and tarsus. No presence of cysts in scleral conjunctiva	102.72	+	125.22	+	+	95
B9	Chronic	Adult	Charolais	Guadalajara	Folds and hyperkeratosis in scrotum, skin of perineum, carpus and tarsus. No presence of cysts in scleral conjunctiva	79.86	+	105.69	+	+	125
B10	Chronic	Adult	Pirenaica	Navarra	Numerous cysts in scrotal skin	136.87	+	81.91	+	+	49
B11	Chronic	Adult	Limousin	Cáceres	Numerous cysts in scrotal skin	54.57	−	54.75	+	+	66
B12	Chronic	59	Avileña	Badajoz	Folds and hyperkeratosis in scrotum, skin of perineum, carpus and tarsus. Presence of cysts in scleral conjunctiva	90.14	+	48.51	+	+	18
B13	Chronic	24	Avileña	Badajoz	Folds and hyperkeratosis in scrotum, skin of perineum, carpus and tarsus. Presence of cysts in scleral conjunctiva	75.92	+	56.02	+	+	58
B14	Chronic	36	Avileña	Badajoz	Folds and hyperkeratosis in scrotum, skin of perineum, carpus and tarsus. Presence of cysts in scleral conjunctiva	79.15	+	94.87	+	+	77
B15	Chronic	23	Avileña	Badajoz	Folds and hyperkeratosis in scrotum, skin of perineum, carpus and tarsus. Presence of cysts in scleral conjunctiva	103.32	+	60.39	+	+	73
B16	Chronic	23	Avileña	Badajoz	Folds and hyperkeratosis in scrotum, skin of perineum, carpus and tarsus. Presence of cysts in scleral conjunctiva	92.32	+	84.63	+	+	64
B17	Chronic	48	Limousin	Madrid	Numerous cysts in scrotal skin	n.a	n.a	n.a	n.a	n.a	n.a

^1^Bull in reproductive age more than 15 months.

+ . positive animal; ELISA, enzyme-linked immunosorbent assay; WB, Western blot; na, not available; RIPC, relative index percent.

All experimental procedures carried out with animals from groups A (*n* = 9) and B (*n* = 3) were approved by the Animal Welfare Committee of the Community of Madrid (Spain) following proceedings described in Spanish and EU legislation (PROEX 92/14, Law 32/2007, R. D. 53/2013) and in Council Directive 2010/63/EU [20, 21]. Males were housed in clinical facilities belonging to the Faculty of Veterinary Sciences of the Complutense University of Madrid (Registration number: ES280790000101).

#### Group A: chronically infected calves with mild lesions in the testis

Group A included nine experimentally infected six-month-old Holstein Friesian male calves [17]. Infected calves were inoculated with 10^6^ bradyzoites via intravenous (*n* = 3), subcutaneous (*n* = 3) and intradermal (*n* = 3) routes. All infected males were seropositive and developed pathognomonic conjunctival cysts from 40 days post-infection (dpi) onwards (Table 1).

#### Group B: non-infected calves

Group B included three non-infected Holstein Friesian male calves that were included in the experimental infection along with the infected calves from group A [17]. All males were seronegative and did not develop any clinical signs or lesions compatible with bovine besnoitiosis (Table 1).

#### Group C: acutely infected bulls with severe lesions in the testicles

Group C consisted of seven acutely infected breeding bulls with systemic clinical signs, severe lesions in the testis and four of them presented aspermia [11] (Bulls 1–7; Table 1). These bulls showed clinical signs or macroscopic lesions compatible with acute besnoitiosis, mainly fever and orchitis, four bulls showed high IgM values in the absence of specific IgG and the remaining male presented high levels of both IgM and IgG antibodies. All males showed severe endothelial injury by marked congestion, thrombosis, necrotizing vasculitis and angiogenesis in the tissues analysed. Vascular lesions coexisted with lesions characteristic of a chronic infection in the majority of bulls. An intense inflammatory infiltrate was also observed in the testicular parenchyma accompanied by different degrees of germline atrophy in the seminiferous tubules with the disappearance of various strata of germ cells in four bulls. Acutely infected bulls included either IgG positive or negative males or males with or without juvenile tissue cysts. For that reason, in order to study more homogeneous and differentiated groups we considered two different categories for the gene expression analyses: acutely infected bulls without tissue cysts and acutely infected bulls with tissue cysts. Bulls without tissue cysts are representative of an early acute infection less than 11–15 dpi based on the chronology of the cyst development [18] and serological results (IgM positive results versus IgG negative results). Bulls with tissue cysts are representative of an acute infection from 11 to 15 dpi onwards until the first month post-infection, and the average cyst diameter varied between 25 and 90 μm [11].

#### Group D: chronically infected bulls with severe lesions in the testis

Group D included ten chronically infected bulls with skin lesions and testis atrophy with azoospermia (Bulls 8–17).

#### Group E: non-infected breeding bulls

Group E consisted of eight non-infected breeding bulls from herds without a previous history of bovine besnoitiosis (Bulls 18–25).

To check the health status of sampled cattle, specific antibodies against relevant cattle pathogens such as *Neospora caninum* and bovine herpes virus-1 were investigated by ELISA techniques (an *in-house* tachyzoite soluble antigen-based ELISA [19] and IDEXX IBR gB X3 Ab Test, IDEXX Inc., USA, respectively). In addition, the presence of bovine viral diarrhoea virus (BVDV) was investigated by antigen detection in sera (IDEXX BVDV Ag/Serum Plus Test, IDEXX Inc., USA).

Blood and testicles (scrotal skin, pampiniform plexus and testicular parenchymal) from all groups were collected and analysed. Samples from groups D and E were collected from regularly slaughtered bulls. The samples were preserved at 4 °C until arrival to the laboratory. Then, blood was centrifuged at 3000 × *g* for 10 min, and serum was preserved at −20 °C until serological analysis to detect specific anti-*B. besnoiti* IgM by ELISA and IgG by conventional ELISA, avidity ELISA and Western blot. Three tissue replicates of scrotal skin, pampiniform plexus and testicular parenchymal were collected from the testis of each male. One sample was frozen at −80 °C for further parasite DNA detection by quantitative PCR (qPCR), another sample was preserved in RNA later buffer (Sigma-Aldrich, Saint Louis, MO, USA) at −80 °C for further host mRNA expression analysis by qPCR, and the last one was stored in 10% buffered formaldehyde for histopathological analysis (conventional histopathology and immunohistochemistry).

In the present study serological analyses, conventional histopathology and PCR analyses were carried out in groups D and E. Immunohistochemistry was accomplished in tissues from groups C (bulls no. 3 and 6), D (bulls no. 9 and 10) and E (bulls no. 20 and 22). mRNA expression analysis was performed in all tissues from experimentally (A and B) and naturally infected groups (C, D and E).

### Parasites and antigen production

Tachyzoites of the in vitro Bb-Spain 1 isolate of *B. besnoiti* were propagated in Marc-145 cell culture monolayers as previously described [22], were cell scraped, purified in cold sterile PBS at a pH of 7.2 using disposable PD-10 desalting columns (GE Healthcare, Chalfont St. Giles, UK) and then pelleted by centrifugation at 1350 × *g* for 10 min at 4 °C. The pellet with tachyzoites was frozen at –80 °C and was used as an antigen source for serological assays and for parasite DNA extraction in PCR techniques. For IgM and IgG BbSALUVET ELISA, 2.0 tachyzoites were lyophilised in a Virtis Benchtop K lyophiliser. Vials for lyophilisation were prepared with 5 × 10^7^ tachyzoites per vial and resuspended in 4 mL of PBS [23].

### Detection of specific antibodies anti-*Besnoitia besnoiti* by serological analyses

#### IgG ELISA

Sera were analysed by conventional BbSALUVET ELISA 2.0 [23] to discriminate between IgG seropositive and seronegative males. A peroxidase-conjugated monoclonal goat anti-bovine IgG (Thermo Fisher Scientific) diluted 1/10 000 in PBST was used as a secondary antibody. The optical density was converted into RIPC using the formula described by García-Lunar et al. [23]. Males with a RIPC ≥ 17.34 were considered positive.

To discriminate between acute and chronic infection, an avidity ELISA was performed when specific IgGs were detected by BbSALUVET ELISA 2.0. The test was carried out as previously described by Diezma-Díaz et al. [17]. Sera were tested using duplicate fourfold dilution series starting from 1:100 to 1:102 400. After incubation with sera, an additional incubation step with 6 M urea was included for one dilution series or with PBS-Tween for the other dilution series. The avidity index (AI) was calculated according to Aguado-Martínez et al. [24], and the cut-off to discriminate between low and high avidity was established at 50.8 according to Schares et al. [25].

#### IgM ELISA

An IgM BbSALUVET ELISA 2.0 was employed following a previously described procedure [20]. Herein, an incubation step with an anti-bovine IgM conjugated with horseradish peroxidase (Bovine IgM Antibody, A10-100P, Bethyl, Mongomery, USA) diluted 1/10 000 in phosphate-buffered saline containing 0.05% Tween 20 (PBST) was included. Control sera used in the ELISA came from an experimental infection carried out in calves [17]. The optical density was converted into the RIPC (relative index percent) using the formula described by García-Lunar et al. [23]. Males with a RIPC ≥ 67.31 were considered positive.

#### Western blot analysis

SALUVET tachyzoite-based Western blot (WB) was performed under non-reducing conditions in 12.5% polyacrylamide gels [26] to confirm the BbSALUVET ELISA 2.0 results [23]. Three main antigenic reactivity areas were described as follows: area I (72.5, 58.9 and 51.4 kDa), area II (38.7, 31.8 and 28.5 kDa) and area III (23.6, 19.1, 17.4 and 14.5 kDa). The recognition of at least three bands in at least two of the three described antigenic areas was considered an IgG positive result [26].

### Parasite DNA detection by conventional (PCR) and quantitative real-time PCR (qPCR)

The DNA extraction of the different tissues collected (testicular parenchyma, pampiniform plexus and scrotal skin) was carried out using the Maxwell® 16 Instrument (Promega, Wisconsin, USA) with the Maxwell® 16 Tissue DNA Purification Kit (Promega, Wisconsin, USA) [27]. The DNA from each sample was quantified by spectrophotometry (NanoDrop, Thermo Scientific; Abs 260/280 nm ratio) and adjusted to 40 ng/μL.

*Besnoitia* spp. DNA was detected by ITS-1 rDNA PCR [28]. The cycling conditions were 2 min at 95 °C, 45 cycles of denaturation at 94 °C for 30 s, annealing at 58 °C for 30 s and extension at 72 °C for 1 min, followed by a final 15 min extension step at 72 °C and maintenance at 4 °C at the completion of the profile. The forward primer ITS1F (5′-TGACATTTAATAACAATCAACCCTT-3′) and the reverse primer ITS1R (5′-GGTTTGTATTAACCAATCCGTGA-3′) were added at a concentration of 10 μM, and the remaining reagents were incorporated into the mixture, as indicated by Frey et al. [27].

The amplified products were visualized after electrophoresis on a 1.5% agarose gel containing 0.1 μL/mL GelRed™ Nucleic Acid Gel Stain (Biotium, USA). DNA extraction and PCR were performed in separate laboratories under biosafety level II conditions (BIO II A Cabinet, TELSTAR, Spain) to avoid cross-contamination. DNA extracted from in vitro cultured *B. besnoiti* Bb-Spain 1 tachyzoites and PCR grade water were used as the positive and negative controls, respectively.

The qPCR assay for the quantification of *Besnoitia* spp. DNA was performed according to Cortes et al. [28] and Frey et al. [27]. The forward primer Bb3 (5′-CAA CAA GAG CAT CGC CTT C-3′) and the reverse primer Bb 6 (5′-ATT AAC CAA TCC GTG ATA GCA G-3′) were added at a concentration of 20 μM, and the remaining reagents were incorporated into the mixture, as indicated by Frey et al. [27]. In each PCR, tenfold serial dilutions of genomic DNA corresponding to 0.1–10 000 Bb-Spain 1 tachyzoites were included. To quantify the amount of DNA, dilutions of DNA extracted from the liver of a cow corresponding to 100, 20, 4, and 1 ng/μL were included. The cycling conditions were 10 min at 95 °C followed by 40 cycles of 95 °C for 15 s and 60 °C for 1 min. Fluorescence emissions were measured during the 60 °C step. A dissociation stage was added. The threshold cycle values (Ct-values) obtained for positive samples were also expressed as tachyzoites per reaction using the standard curve that was included in each run as indicated by Frey et al. [27]. Only *Besnoitia* PCR positive samples were further analysed by qPCR.

### RNA extraction, cDNA synthesis and host gene expression

Quantitative PCR was used in order to study the mRNA expression levels of pro-inflammatory cytokines (IL-1α, IL-6, IL-8, IL-17A, and TNF-α), anti-inflammatory/regulatory cytokines (IL-4, IL-10, TGF-β1), chemokines (CCL2, CCL24, and CXCL2), leukocyte adhesion molecules (ICAM1, VCAM1, and SELE), pattern recognition receptors (PRRs) (TLR2), plasminogen activator, tissue type (PLAT) and genes related to extracellular matrix (ECM) remodelling (ADAMTS1, MMP-13, TIMP-1 and SERP-1) in scrotal skin, testicular parenchyma and pampiniform plexus from all animals included in this study. The primers employed in the qPCRs are shown in Additional file 1. The RNA extraction of the different tissue samples was completed using the TRIzol method (TRIzol Reagent, Invitrogen). Briefly, 50 mg of each tissue was homogenized in 1 mL of TRIzol reagent per 100 mg of tissue using a tissue homogenizer (Kinematica™ Polytron™ PT 1600 D). Post-centrifugation, RNA was extracted with chloroform and precipitated with isopropyl alcohol. RNA samples were then re-suspended in 30 μL of DEPC treated H_2_O. For all samples, RNA concentrations were determined by spectrophotometry (Nanophotometer, Implen), and the integrity of the RNA was checked by the 260/280 absorbance ratio (close to 2.0) and the visualization of the 18S and 28S ribosomal subunits after electrophoresis on a 1% agarose gel. Reverse transcription was performed using the master mix SuperScript® VILO™ cDNA Synthesis Kit (Invitrogen, Paisley, UK) in a 20 μL reaction using up to 2.5 μg of total RNA. Quantitative real-time PCR was performed in 25 μL volumes using 12.5 μL of Power SYBR®PCR Master Mix (Applied Biosystems, Foster City, CA, USA), 10 pmol of each primer and 5 μL of the diluted cDNA sample. Reactions were performed in an ABI 7500 FAST Real Time PCR System (Applied Biosystems, Foster City, CA, USA) with the following amplification conditions: 95 °C for 10 min followed by 40 cycles at 95 °C for 15 s and 60 °C for 1 min. For each target gene, a seven-point standard curve was included in each batch of amplifications based on tenfold serial dilutions starting at 10 ng/µL plasmid DNA, in which the full-length cDNA containing the gene fragments used as templates in qPCR were cloned. The relative expression was calculated using the comparative method 2^−ΔΔ*C*T^ [29] after normalization with the housekeeping genes actin β (ACTB) (ENSBTAG00000026199) and separately GAPDH (ENSBTAG00000014731) for bovine genes [16].

### Histopathology

Routine paraffin wax embedding procedures were used. Tissue samples from testicular parenchyma, pampiniform plexus and scrotal skin were fixed in 10% buffered formaldehyde, dehydrated in a graded ethanol series, and cleared in xylene. The samples were embedded in paraffin wax, and sections with a thickness of 5 μm were cut using a sliding microtome (Leica Microsystems, Germany). Sections were stained with haematoxylin–eosin (H&E), periodic acid-Schiff (PAS) and Masson trichrome (for better visualization of connective tissue) and underwent a light microscopy evaluation. Photomicrographs of each studied specimen were subjected to computer-assisted image analysis using a computer coupled to an optical Olympus BX50 microscope equipped with a Colour View IIIu digital Olympus DP27 camera (Olympus, Japan). Parasite cysts found in the histological sections were counted in 10 randomized fields with a 10 × magnification objective to obtain an average number of parasite cysts and to measure the diameter of the cysts.

### Immunohistochemistry

Scrotal skin, testicular parenchyma and pampiniform plexus samples from acutely infected bulls with and without tissue cysts (bulls no. 3 and 6) and chronically infected groups (bulls no. 9 and 10) as well as from two non-infected males (bulls no. 3 and 5) were selected to carry out an immunohistochemical labelling in order to characterize the inflammatory infiltrate. Thus, a panel of different antibodies raised against antigens expressed by T-lymphocytes, B-lymphocytes and macrophages was used for the study of its distribution and proportion (Additional file 2).

Briefly, paraffin sections were placed onto positively charged slides (Flex IHC Slides, Agilent technologies) and deparaffinization and epitope retrieval was carried out by using a pretreatment module for tissue specimens (PT-Link System, Agilent technologies) under different conditions according to the primary antibody (Additional file 2). After blocking endogenous peroxidase by immersion of the sections into a 3% H_2_O_2_ in methanol solution for 30 min in the dark at room temperature, samples were incubated overnight at 4 °C with the primary antibody diluted in PBS. After washing, slides were incubated with the appropriate HRP-labelled polymer secondary antibody (EnVision System, Agilent Technologies). Finally, the reaction was developed at room temperature with 3,3-diaminobenzidine (DAB, Agilent Technologies) producing a brown signal, rinsed in tap water and counterstained with Mayer’s haematoxylin for 10 s.

Isotype-matched immunoglobulins of the appropriate species were used as a control. These included sections with an isotype control for each primary antibody and the omission of the primary antibody. As a positive control, sections from tissue samples processed in the same manner and known to contain the cell population of interest were included with the primary antibodies as positive controls.

The presence and distribution of inflammatory cells were studied in all selected sections from all the tissues mentioned above. In addition, to study the proportions of positive immunolabelled cells between different bulls and groups, a total of 20 pictures were taken under the 40× objective of each section from regions with inflammatory infiltrate. The measurement of the labelled area with the markers employed was carried out by a digital analysis method in which the fractional area labelled positively and referred to as the proportional target area (PTA) was evaluated as previously described by Gutiérrez-Expósito et al. [30]. Image analysis was performed using the ImageJ processing and analysis software (US National Institutes of Health, Bethesda, Maryland, USA).

### Statistical analysis

Messenger RNA expression levels of all the investigated genes in scrotal skin, testicular parenchyma and pampiniform plexus were analysed. Thus, on one hand experimentally infected calves (Groups A and B) were compared independently from bulls. On the other hand, groups C, D and E were also compared. The non-parametric Kruskal–Wallis test, followed by Dunn’s multiple range test, were used for both analyses. The statistical significance for all the analyses was established with *P* < 0.05. GraphPad Prism 6 v.6.01 (San Diego, CA, USA) software was used to perform all the statistical analyses and create all of the graphical illustrations.

## Results

As mentioned above a summary of the most relevant histopathological findings (lesions and tissue cysts) together with serological and molecular (DNA detection) results previously published for groups A, B and C are shown in Table 1.

### Serological results in groups D and E

All chronically infected bulls (group D) analysed were seropositive by both ELISA and Western blot and showed high avidity index (Table 2) except for one bull that was seronegative by IgM-based ELISA and two bulls with a low avidity index. Neither specific IgM nor IgG were detected in non-infected bulls (group E).

### Parasite DNA detection in groups D and E

*Besnoitia* spp. DNA was present in all tissues (*n* = 30) analysed from chronically infected bulls and was quantified in twenty-eight positive PCR tissues (Table 3). The highest parasite burden was found in the scrotal skin and testicular parenchyma compared to pampiniform plexus (*P* < 0.001 and *P* < 0.01). The presence of *Besnoitia* spp. DNA was not detected in any calf from group E.

**Table 3:** ***Besnoitia*** **spp. detection by means of PCR and histological techniques in testicle tissues of chronically infected bulls**

Bull	Pampiniform plexus			Testicular parenchyma			Scrotal skin		
	PCR	qPCR (zoites/mg tissue)	Histology (mean cysts diameter (µm)	PCR	qPCR (zoites/mg tissue)	Histology (mean cysts diameter (µm)	PCR	qPCR (zoites/mg tissue)	Histology (average cysts diameter; µm)
8	POS	4.5 × 10^–5^	272.71	POS	3.66	–	POS	2 × 10^–5^	232.74
9	POS	6.4 × 10^–5^	225.00	POS	51.12	225.00	POS	4017.92	253.17
10	POS	nd	150.09	POS	9.7 × 10^–1^	–	POS	nd	121.08
11	POS	1.2 × 10^–4^	196.76	POS	393.09	189.46	POS	13,442.67	316.91
12	POS	1.3 × 10^–1^	142.43	POS	8.08	–	POS	3818.31	183.90
13	POS	1.2 × 10^–1^	133.96	POS	2.4 × 10^–1^	–	POS	2130.38	210.69
14	POS	1 × 10^–4^	171.44	POS	1.09	168.43	POS	1.6 × 10^–2^	236.38
15	POS	9.5 × 10^–2^	126.41	POS	3.3 × 10^–1^	–	POS	7.7 × 10^–3^	252.69
16	POS	9.7 × 10^–3^	174.56	POS	4.2 × 10^–1^	–	POS	1899.65	235.59
17	POS	8.6 × 10^–2^	176.95	POS	11.45	–	POS	3017.55	327.69

POS, detection of *Besnoitia* spp. DNA; nd, not determined; –,cysts not found.

### Histopathological findings in the testes from groups D and E

#### Tissue cysts in testes from chronically infected bulls

**Figure 1:**
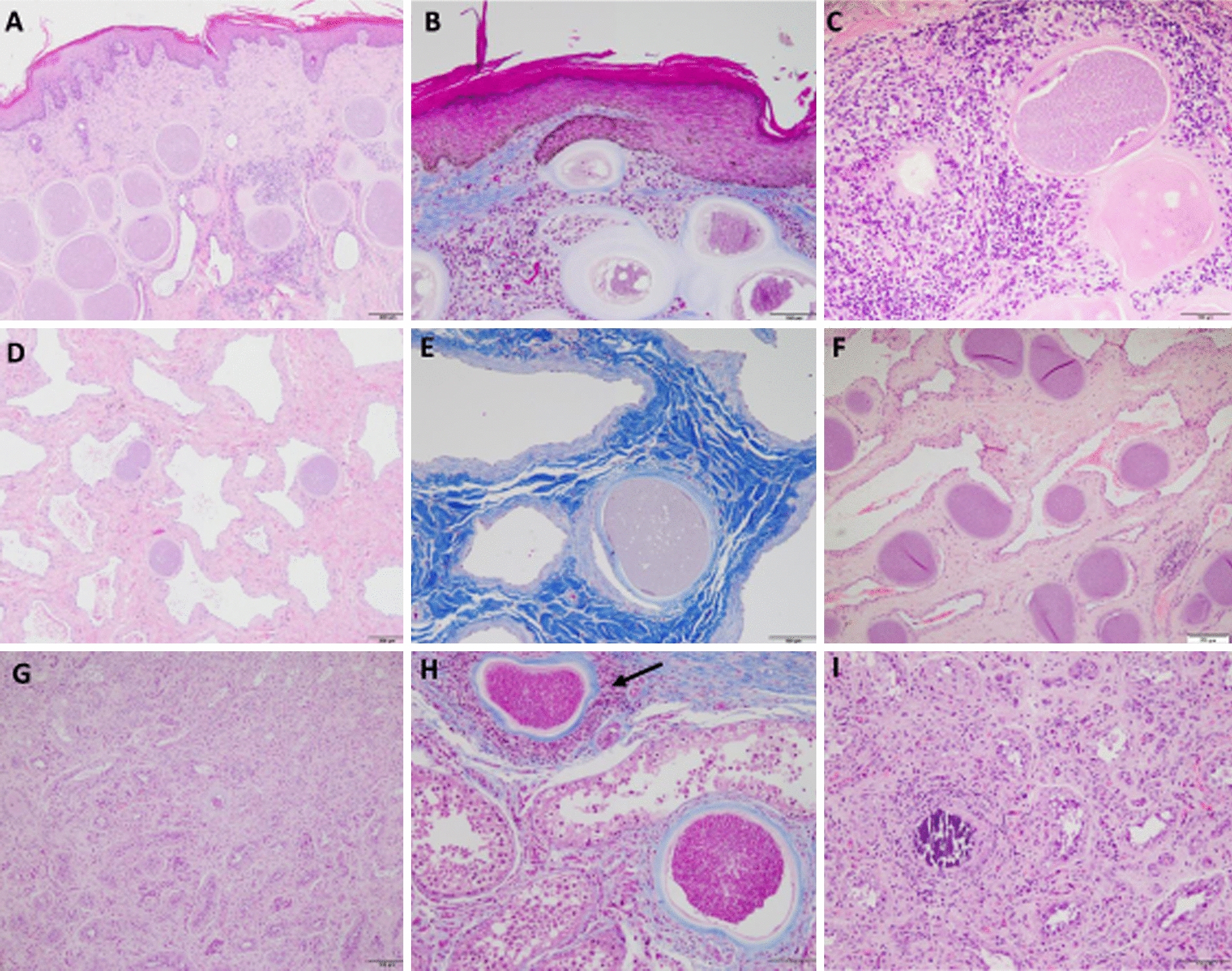
**Microscopic lesions found in the scrotal skin, pampiniform plexus and testicular parenchyma tissue sections from chronically infected bulls.** Lesions were visualized by either haematoxylin–eosin (H-E) or Masson’s trichrome (M-T) staining. **A** (H-E) Hyperkeratosis, acanthosis and dilated sweat glands associated with tissue cysts in the scrotum (bull no. 17); **B** (M-T) Hyperkeratosis and abundant fibrosis associated with tissue cysts in the scrotal skin (bull no. 12); **C** (H-E) Hyperkeratosis and numerous tissue cysts surrounded by connective tissue and abundant inflammatory infiltrate in the scrotal skin (bull no. 16); **D** (H-E) Tissue cysts in the lumen of the vessels associated with the scarce inflammatory reaction in the pampiniform plexus (bull no. 17); **E** (M-T) Abundant fibrosis associated with tissue cysts in the pampiniform plexus (Masson’s trichrome stain) (bull no. 17); **F** (H-E) Higher number of tissue cysts in the lumen of the vessels associated with the scarce inflammatory reaction in the pampiniform plexus (bull no. 8) G**:** (H-E) Degenerated seminiferous tubules and absence of sperm (bull no. 10); **H** (M-T) Tissue cysts with fibrosis and the inflammatory reaction in one of them (black arrows) and seminiferous tubules with different degrees of atrophy inside (Masson’s trichrome stain) (bull no. 13); **I** (H-E) Tissue cysts necrotic with inflammatory infiltrate, degenerated seminiferous tubules and the absence of sperm and fibrosis (bull no. 13). Scale bars: **A**, **C**, **D**, **F**, **G**: 200 µm; **B** and **E**: 100 µm.

Mature tissue cysts were always detected regardless of the tissue analysed. Tissue cysts were present in the scrotal skin of all chronically infected bulls, nine out of ten bulls presented tissue cysts in pampiniform plexus and seven bulls presented scarce tissue cysts in the testicular parenchyma. These results contrast with the higher parasite load detected by qPCR in the testicular parenchyma compared with the pampiniform plexus, which can be explained by the low pampiniform plexus tissue integrity as indicated by the low yield DNA extraction (data not shown). The average tissue cyst diameters were 177.03, 237.08 and 194.29 μm in the pampiniform plexus, scrotal skin and testicular parenchyma, respectively, being significantly higher in the scrotal skin than in the pampiniform plexus (*P* = 0.0462) (Figure 1).

Tissue cysts in scrotal skin were surrounded by an intense inflammatory reaction with a predominance of macrophages that acquire a palisade disposition around the cyst, accompanied by multinucleated giant cells and a few eosinophils (Figures 1A–C). Granulomatous reactions (lymphocytes, plasma cells and macrophages) with small central necrotic foci were also observed that could be compatible with a reminiscent degenerated parasitic cyst in bulls no. 8 and 9.

Fewer tissue cysts appeared in the pampiniform plexus, and normally isolated cysts were the most predominant finding. They were occasionally observed in the connective tissue of the pampiniform plexus and in most cases in a subendothelial localization in the vascular walls, with hardly any inflammatory infiltration. They usually occluded the vascular lumen (Figures 1D–F).

Scarce tissue cysts were found in the subendothelial localization in the vascular walls and in the connective tissue in the testicular parenchyma.

#### Lesions

Similar lesions were present in all tissues that included vascular lesions, inflammatory infiltrate, fibrosis and testicular degeneration. Diffuse thickening of the stratum corneum (hyperkeratosis) and an increase in the spiny layer (acanthosis) were observed in the epidermis of the scrotal skin in all bulls (Figure 1A). Additionally, marked ecstasies of the sweat glands were also evident in all bulls (Figure 1A). Vascular injury was detected in all tissues and was more intense in the scrotal skin. In particular, angiogenesis and necrotizing vasculitis were observed in the blood vessels of the testicular parenchyma, tunica albuginea, pampiniform plexus and scrotal skin. Inflammatory infiltrate was present in all tissues, most predominantly in the scrotal skin followed by the pampiniform plexus and the testicular parenchyma. Some vessels had lymphocytic infiltrates in the wall (vasculitis). Hyalinosis and thickening of the basement membrane were also observed. An intense lymphoplasmocitary infiltrate was observed in scrotal skin with abundant connective tissue, mainly collagen, the presence of macrophages, marked proliferation of fibroblasts and interstitial fibrosis (Figure 1B). Scarce to moderate lymphoplasmacytic infiltrates with the presence of macrophages, abundant granulation connective tissue, fibroblasts and collagen fibres (moderate to intense fibrosis) and some siderocytes were observed in the pampiniform plexus (Figures 1D–F). An intense inflammatory infiltrate with the presence of lymphocytes, some macrophages, abundant young granulation tissue, with an abundance of fibroblasts, and collagen were present in the testicular parenchyma of four bulls (bulls no. 13, 15, 16 and 17). In two males (bulls 12 and 14), an increase in elongated cells of fibroblastic nature, an abundance of collagen, a scarce infiltration of round cells and macrophages, and a relative hyperplasia of Leydig cells were observed in the interstice. Remarkably, all cattle presented aspermia with different degrees of germline atrophy in the seminiferous tubules with the disappearance of the various layers of spermatids to spermatocytes (various degrees of testicular degeneration) as well as degenerated seminiferous tubules (Figures 1G and I). Sertoli cell-only syndrome, focal areas of tubular necrosis and small dystrophic calcifications were observed in three males (bulls 9, 15 and 17) (Figure 1I). In four bulls (no. 11, 12, 13 and 15) spermatids were not observed, and the seminiferous tubes structure was not distinguished.

### Immunohistochemistry in tissues from naturally infected bulls (Groups C, D and E)

The inflammatory infiltrate found in the scrotal skin, pampiniform plexus and testicular parenchyma was characterized in acutely (Group C: bulls no. 2 and 6) and chronically infected bulls (Group D: bulls no. 10 and 12) and compared with the same tissues from healthy bulls used as non-infected controls (Group E: bulls no. 20 and 22). In this sense, CD3 + and Iba1 + cells were found in all bulls, including non-infected bulls, MAC387 + cells were only detected in infected bulls and CD21 + cells were not present or rarely observed in all bulls except for the scrotal skin of one acutely infected bull without tissue cysts. In non-infected bulls, CD3 + cells were restricted to scrotal skin samples with a focal distribution in a few isolated foci of inflammation. However, Iba1 + cells were detected in all tissue samples from both non-infected bulls. Specifically, the distribution of scarce Iba1 + cells in scrotal skin and pampiniform plexus from non-infected bulls was homogeneous over the tissue, whereas in testicular parenchyma, they were restricted around seminiferous tubules.

**Figure 2:**
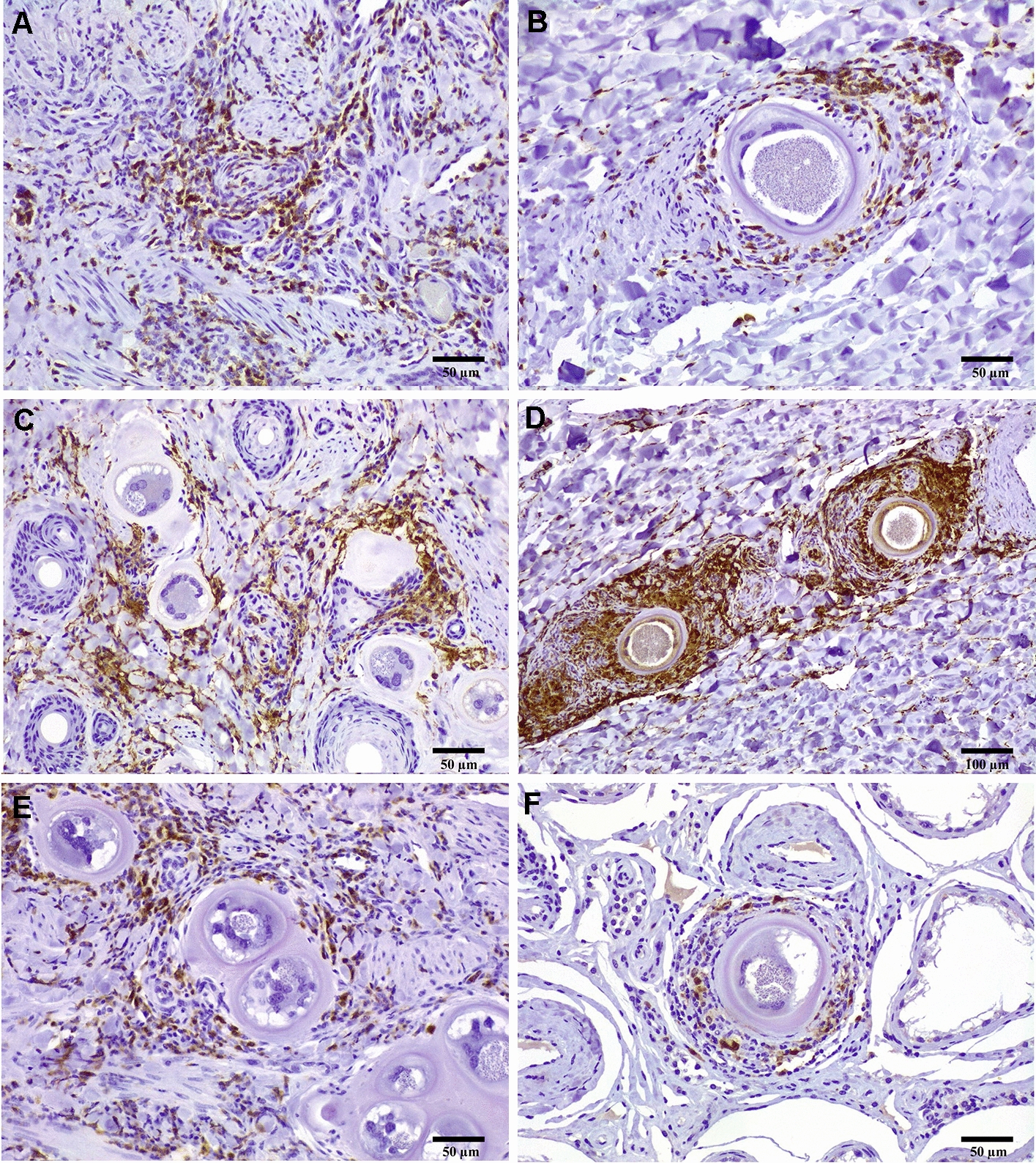
**Immunohistochemistry in the scrotal skin, pampiniform plexus and testicular parenchyma from acutely and chronically infected bulls.** **A** Widespread presence of T-lymphocytes (CD3 + cells) associated with blood vessels in the scrotal skin from a acutely infected bull with juveniles cysts (bull no. 6); **B** CD3 + T-lymphocytes surrounding tissue cysts in the pampiniform plexus from a chronically infected bull (bull no. 10); **C** Diffuse presence of macrophages (iba-1 + cells) in focal areas of vasculitis and surrounding juvenile tissue cysts in the scrotal skin of an acutely infected bull (bull no. 3); **D** iba-1 + macrophages surrounding tissue cysts without diffuse inflammation in the pampiniform plexus of a chronically infected bull. (bull no. 10); **E** CD3 + T-lymphocytes surrounding tissue cysts in the scrotal skin from a chronically infected bull (bull no. 10); **F** Immunohistochemical detection of several active macrophages MAC387 + surrounding tissue cysts in the testicular parenchyma from a chronically infected bull (bull no. 10).

Regarding infected bulls, numerous inflammatory infiltrates were observed in the scrotal skin, pampiniform plexus and testicular parenchyma from acutely and chronically infected bulls and were characterized by the presence of T-lymphocytes (CD3 + cells) and macrophages (Iba1 + /MAC387 + and Iba1 + /MAC387- cells) together with the absence of B-lymphocytes (CD21 + cells) (Figure 2). The presence and distribution of these inflammatory infiltrates were extremely variable between tissues and stages of the disease; and, even within the same section, we found tissue cysts with and without inflammatory infiltrate.

CD3 + cells were detected in all tissues and stages except for the pampiniform plexus of bull no. 6 (acutely infected bull without *Besnoitia* spp. tissue cysts), where several inflammatory foci with CD3 + cells were associated with blood vessels from the testicular parenchyma and scrotal skin (Figure 2A). The distribution of CD3 + cells was diffuse, although a higher number of CD3 + cells was observed surrounding tissue cysts, above all in bull no. 3 (acutely infected bull with tissue cysts), while surprisingly, the presence of CD3 + cells around the tissue cysts from chronically infected bulls was reduced (Figures 2B and E). Regarding PTA, the expressions of CD3 in scrotal skin in acutely (7.2%) and chronically (7.4%) infected bulls were higher than in controls (4.14%) and extremely variable between infected bulls in the pampiniform plexus (0–7.3%) and testicular parenchyma (2.4%—6.76%). In fact, the highest and lowest levels of CD3 expression were both observed in chronically infected bulls.

Otherwise, regarding the presence and distribution of macrophages, there was a clear predominance of Iba-1 + cells over MAC387 + cells in all infected bulls, both observed mainly surrounding tissue cysts. Nevertheless, macrophages were also found in the focal areas of vasculitis in both acutely infected bulls, mainly in the scrotal skin (Figure 2C). In addition, when juvenile tissue cysts were present, diffuse inflammation was observed together with a pronounced inflammation close to them in all tissues. In addition, macrophages observed in acutely infected bulls were Iba1 + /MAC387- except for the scrotal skin from bull no. 6 without tissue cysts and testicular parenchyma from bull no. 3 with tissue cysts (Iba1 + /MAC387 +). In contrast, all macrophages present in all tissues from chronically infected bulls were exclusively located surrounding tissue cysts (Figures 2D and F). In this sense, one chronically infected bull showed Iba1 + /MAC387- macrophages and the other one showed Iba1 + /MAC387 + macrophages, demonstrating that the latter had a higher degree of inflammation in all tissues. Regarding PTA (see Additional file 3), the expression of Iba1 was higher in tissues from chronically (12% on average) compared with acutely (6% on average) infected bulls and always lower than in controls (3.3%). Remarkably, the highest expression of Iba1 was observed in the pampiniform plexus (18.9%) and testicular parenchyma (17.1%) from one of the chronically infected bulls. Nevertheless, the proportion of MAC387 + varied from 2.71% to 7.88% and from 1.87% to 4.34% in tissues from acutely and chronically infected bulls, respectively, demonstrating that the testicular parenchyma had the highest PTAs in both stages.

### Gene expression levels in tissues from experimentally infected calves (groups A and B)

In experimentally infected calves, gene expression levels were similar in all tissues and in all infected groups.

Gene expression values of differentially expressed genes (*P* < 0.05) in the three tissues investigated are shown in Table 4 and Additional file 4. There was a higher gene regulation in the intradermally inoculated group, followed by the subcutaneously inoculated group and finally the intravenously inoculated group. The tissue that showed the highest gene regulation was the scrotal skin, followed by the pampiniform plexus and the testicular parenchyma. Most genes were downregulated, except for CCL24 and CXCL2 genes, which were upregulated. Furthermore, most differentially expressed genes showed similar up or down regulation for each tissue regardless of the inoculation group (Table 4).

**Table 4:** **Differentially expressed genes in testicle tissues of experimentally and naturally infected males**

Analyzed tissue	Gene regulation	Intravenously inoculated group	Subcutaneously inoculated group	Intradermally inoculated group
Experimentally infected calves				
Scrotal skin	Upregulation	CCL24	No significant changes	No significant changes
	Downregulation	IL10; TLR2	IL6; IL8; IL10; SERP; SELE; PLAT; ICAM1; ADAMTS1; IL4; IL17; TGF; TNF	IL6; IL8; IL10; IL1a; TLR2; SELE; PLAT; ICAM1; VCAM1; CCL2; CXCL2; TIMP1; ADAMTS1; IL17; TGF; TNF
Pampiniform plexus	Upregulation	CCL24	No significant changes	CCL24
	Downregulation	IL6; ADAMTS1	IL6; IL8; IL10; IL1a; SERP; PLAT; CCL2; ADAMTS1; TIMP1; IL17	IL6; IL8; IL10; IL1a; TLR2; SERP; SELE; PLAT; ICAM1; VCAM1; CCL2; TIMP1; ADAMTS1; IL17; TGF; TNF
Testicular parenchyma	Upregulation	No significant changes	CXCL2	CCL24
	Downregulation	IL6; IL8; IL10; SERP; CCL2; ADAMTS1; TIMP1	IL6; IL8; SERP; PLAT; CCL2; ADAMTS1; IL17; TGF	IL6; IL8; IL10; SELE; PLAT; VCAM1; TIMP1; IL17; TLR2; TNF; TGF

Analyzed tissue	Gene regulation	Acutely infected bulls without tissue cysts	Acutely infected bulls with tissue cysts	Chronically infected bulls
Naturally infected animals				
Scrotal skin	Upregulation	No significant changes	No significant changes	ICAM1
	Downregulation	No significant changes	No significant changes	PLAT; IL8
Pampiniform plexus	Upregulation	PLAT; IL1a	PLAT; TLR2	ICAM1
	Downregulation	No significant changes	No significant changes	PLAT; MMP13
Testicular parenchyma	Upregulation	PLAT; IL1a; MMP13; TIMP1; IL6	IL1a; TIMP1	ICAM1
	Downregulation	IL8; IL4; CCL24	MMP13; IL8; CCL24	PLAT; IL8; MMP13; IL1a; IL6; IL4; TGFB1; CCL24

### Gene expression in tissues from naturally infected bulls (Groups C, D and E)

Our result showed gene regulation mainly in the testicular parenchyma in both acute and chronically infected bulls.

**Figure 3:**
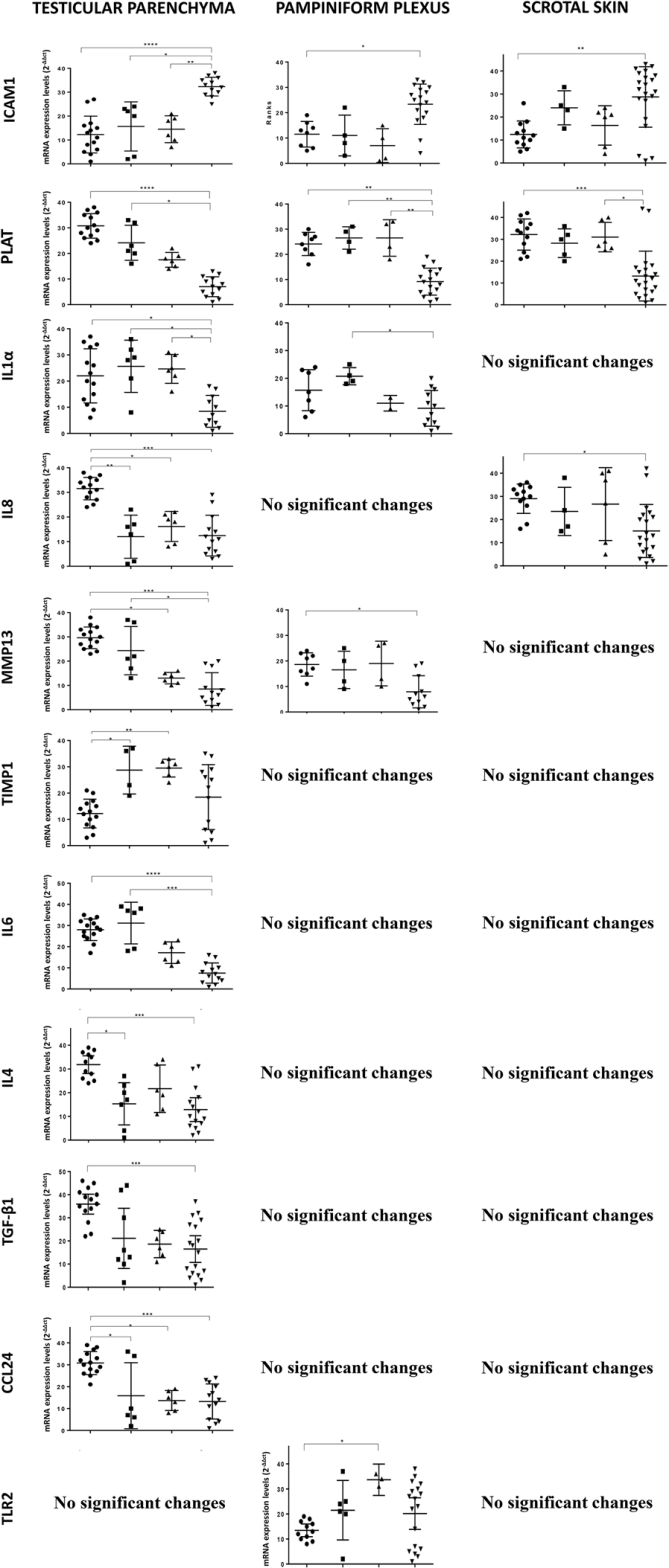
**Transcripts that showed significant variation in gene expression in the testicles from bulls.** Non-infected bulls (●), acutely infected bulls without tissue cysts (■), acutely infected bulls with tissue cysts (▲) and chronically infected bulls (▼). Data are presented as individual points. Horizontal lines represent median values with the 95% CI for each group. ****, ***, ** and * indicate *P* < 0.0001, *P* < 0.001, *P* < 0.01 and *P* < 0.05 significant differences, respectively.

Differentially expressed genes in the three tissues investigated are shown in Table 4 and Figure 3. IL-10, IL-17, ADAMTS1, VCAM1, SELE, TNF and CCL2 did not show significant variations among groups (data not shown).

Higher gene regulation was observed in the testicular parenchyma, followed by the pampiniform plexus and finally the scrotal skin in the three groups studied. Upregulated genes predominated in the testicular parenchyma of acutely infected bulls without tissue cysts, whereas downregulated genes were more numerous in the same tissue from chronically infected bulls. IL-8, IL-4 and CCL24 were downregulated in the testicular parenchyma regardless of the group studied. In contrast, different gene regulation kinetics were observed for IL-1α and PLAT genes that were upregulated in acutely infected bulls followed by downregulation in chronically infected bulls (Table 4, Figure 3).

## Discussion

Herein we have studied a wide panel of molecular biomarkers in testicles from males experimentally and naturally infected with *B. besnoiti*. The results indicated that a few biomarkers were associated with acute, chronic or subclinical bovine besnoitiosis in males. These biomarkers could reflect the coexistence of vascular lesions, inflammation and fibrosis found in all tissues with variable degrees of severity and could be related with an intense macrophage infiltrate found in the testicular parenchyma. A few genes have been identified as markers of infection and the prognosis of sterility.

The cattle included in the study were carefully selected. A restrictive selection criterion included a combination of clinical, pathological, serological and molecular results. First, testicles from *B. besnoiti* experimentally infected male calves were analysed. These males belonged to a recent study where a chronic model of bovine besnoitiosis was developed by the inoculation of the bradyzoite stage. As these animals developed mild to moderate clinical signs, mild lesions, tissue cysts and presented a normal seminiferous epithelium structure, they were considered as representative of a subclinical infection [17, 21]. This group is relevant since in areas where the disease is endemic, most clinical cases are usually unapparent or with no relevant clinical signs and lesions [31]. Second, a panel of sterile and severely affected bulls with either acute or chronic besnoitiosis were selected. This study included the highest number of severely infected and sterile bulls studied to date [8, 32]. It was recently reported that severe acute besnoitiosis leads to early sterility with a predominance of severe vascular injury and marked inflammatory response [11]. Regarding the histological lesions found in chronically infected bulls, all males were similarly affected. Most lesions have been previously reported by the few studies published [12, 32]. In this context, there are similarities with previous studies, such as the presence of tissue cysts in the pampiniform plexus, fibrosis, alteration of spermatogenesis, skin lesions in the scrotum and inflammatory infiltrates around the tissue cysts. However, there are interesting findings that were common to all bulls and that had not been described or at least not described in so many animals including the following: coexistence of vascular lesions, inflammatory infiltration in the parenchyma indicated by immunohistochemistry and the presence of tissue cysts in the testicular parenchyma, which could be correlated with the severity of the disease and the consequences at the reproductive level.

Despite the undoubted value of the bulls and calves samples, we identified limitations that hampered the identification of a higher number molecular markers. First, a low number of experimentally infected calves per group could be analysed, and the immature immune system of calves [33] may have also hampered the identification of markers, as we have hypothesized that macrophages followed by T-cells might play a crucial role in the pathogenesis. Second, the infection progress differed among the studied field cattle. To study more homogeneous groups, we considered two different categories of acutely infected bulls: bulls without tissue cysts and bulls with tissue cysts. However, the differences observed in the tissue cyst diameter were indicative of the variable post-infection time [11]. In addition, chronically infected bulls also displayed differences. Individual variability was also shown by IgG avidity results since two males (no. 10 and 12) showed low avidity values indicative of a recent infection, and these results were supported by the smaller tissue cyst size detected in the scrotal skin of these bulls, which is compatible with an early chronic infection. We found scrotal mature cysts with an average size that varied from 121–184 μm up to tissue cysts exceeding 300 μm. It is difficult to estimate with precision the time point post-infection since the tissue cyst size is not only influenced by the progression of the infection but also by both peripheral and local host-immune responses that could have limited the growth of the tissue cysts [21].

The gene regulation found in this study was different in naturally infected bulls compared to the experimentally infected calves, and it may be explained by the different stages of disease progression, severity and parasite load. In experimentally infected calves, gene regulation seems to be associated with the presence of lesions and a higher parasite load, as scrotal skin from intradermally inoculated calves that showed a higher number of lesions and was more parasitized [21], presented the highest number of differentially expressed genes (DEGs). There was a predominance of downregulated genes (e.g., IL-6, IL-1α, SELE, ICAM1, ADAMTS1 and CCL2) in contrast to the upregulation evidenced in an in vitro model, where many upregulated genes were thought to be associated with endothelial activation [14]. The absence of a pro-inflammatory phenotype could be explained by the absence of severe injury in a subclinical infection, where populations of anti-inflammatory M2 macrophages predominate at least in the testicular parenchyma [34] and could modulate the pathways activated during the initial phases of endothelial injury. The CCL24 gene was consistently upregulated and could indicate a chemotactic activity for eosinophils at the focus of inflammation, preparing them for the release of TNFa, TGF-β and IL-6 involved in the microvascular leakage [35].

In naturally infected bulls, the testicular parenchyma was the tissue that showed the highest gene regulation, probably associated with the presence of an intense inflammatory infiltrate (Table 4, Figure 3). The results obtained in acutely infected bulls agree with previous studies performed in vitro in endothelial cells [13, 14], as pro-inflammatory cytokines (IL-6 and IL-1α), PLAT and extracellular matrix-related genes (MMP13, TIMP1) were upregulated in the pampiniform plexus and testicular parenchyma with severe vascular lesions (e.g., vasculitis and thrombosis) and intense inflammatory infiltrate, as described by González-Barrio et al. [11].

These genes have been associated with vascular damage, as they can act as inflammatory mediators triggering an antifibrinolytic-coagulation cascade that activates clotting and the development of a provisional extracellular matrix (ECM), exemplified by fibrinolysis pathways. In addition, platelet activation and degranulation promote blood vessel dilation and permeability, allowing efficient recruitment of inflammatory monocytes at the site of tissue injury, also increasing oedema. In a previous study, coagulation-related genes, specifically in the fibrinolysis pathway, were modulated by *B. besnoitia* in vitro infection [14], with the upregulation of several molecules with fibrinolytic properties such as plasminogen activator, tissue type (PLAT) at 12 h post-infection (hpi). In our study, we observed the upregulation of the PLAT gene in all tissues. This finding could be associated with the initial leukocyte attraction to the site of endothelial damage, and macrophages are likely to play a key role. Indeed, in acutely infected bulls, pro-inflammatory cytokines are present together with Iba-1 + /MAC387 + macrophages. Testicular macrophages comprise the largest immune cell population in the mammalian testis, and they are characterized by an attenuated pro-inflammatory response upon adequate stimulation. However, during infection the immune response characteristics of testicular macrophages reflect the need to maintain a balance between a strong reaction to counter invasive pathogens and preventing excessive levels of pro-inflammatory cytokines with the potential to disrupt or destroy spermatogenesis [34]. In this context, through immunohistochemistry we can see how the populations of iba1 + macrophages are more numerous in infected bulls and are also present in non-infected males, suggesting that these macrophages could be the resident populations. However, other macrophage populations such as MAC387 + are only present in infected males, especially in testicular parenchyma, which could indicate that these macrophages have reached the focus of inflammation via the blood circulation (circulating macrophages). Despite some studies confirming the role of circulating macrophages in testicular inflammation, the role of macrophages derived from blood monocytes is under-researched [34], and these macrophages could play an important role in the testicular degeneration or azoospermia that occurs in infected bulls. Activated macrophages (M1 cells) can further exacerbate the inflammatory response by recruiting large numbers of T helper cells, such as T-lymphocytes (CD3), as we can see in the tissues of infected bulls (pampiniform plexus and testicular parenchyma), both acute and chronically infected, whose values are higher than in non-infected bulls.

When parasite tissue cysts develop, M1 cell activation might be diminished, and TH2-type cytokines could drive the immune response towards a tissue healing response, which is characterized by the accumulation of M2 macrophages that promote tissue healing and fibrosis through the production of MMPs and tissue inhibitor of metalloproteinases 1 (TIMP1). Studies have suggested that progressive fibrotic diseases, such as idiopathic pulmonary fibrosis, hepatic fibrosis and systemic sclerosis, are highly regulated by macrophages [36]. Tissue remodelling is the result of an imbalance in the equilibrium of the normal processes of synthesis and degradation of ECM components markedly controlled by MMPs/TIMP imbalance. The activities of MMPs that can degrade matrix might be under-expressed in fibrosis or, if present, could function to resolve the excess matrix [37]. In agreement with this assumption, in our study the MMP13 gene was downregulated in the pampiniform plexus and testicular parenchyma from males with tissue cysts (both acute and chronic infection) compared to the negative group. The expression of the MMP-13 gene in other chronic diseases such as liver cirrhosis has been very limited during the recovery phase; however, in the early phase of liver cirrhosis, the transient overexpression of MMP-13 mRNA is induced in macrophages associated with liver scarring during the spontaneous regression of liver fibrosis (hepatic fibrogenesis) [37]. Similarly, in the testicular parenchyma from males with early infection (acute infection without tissue cysts), we observed that MMP-13 gene expression was upregulated compared to males with chronic infection. Previous studies in the closely related parasites *T. gondii* and *N. caninum* have shown a high upregulation of MMP-13, which may be important for crossing biological barriers and intra-organic dissemination [16]. TIMPs are endogenous protein regulators of the MMP family. Increased levels of TIMP lead to the accumulation of ECM, while the loss of TIMP leads to increased proteolysis of the matrix [38]. In our study, we observed a significant upregulation of TIMP1 in the testicular parenchyma from acutely infected bulls compared to negative controls.

Other markers probably produced by pro-fibrotic macrophages, which are usually genes related to the early stages of infection, have also been described in chronic infection pathways. As an example, the PLAT gene plays an important role in cell migration and tissue reconstruction and is active in degrading ECM components [39]. In our study, the PLAT gene was down regulated in all tissues from chronically infected bulls compared to non-infected bulls. In several studies, PLAT expression was mainly inhibited by plasminogen activator inhibitor-1 (PAI-1), and it is suggested that PAI-1 promotes pathological fibrosis [39, 40] and also regulates ICAM1 by increasing its expression [41]. Our results are in agreement with these previous works since the PLAT gene was downregulated and the ICAM-1 gene was upregulated in chronically infected animals. ICAM-1 would play an important role in neutrophil and lymphocyte trafficking and would act as an accessory molecule in antigen presentation. Several studies have pointed to a role of ICAM-1 expression in the pathogenesis of chronic diseases where fibrosis is present [41, 42].

When gene expression was studied in the different tissues from naturally infected bulls, several selected genes did not show significant variations, which can probably be explained by two main reasons. First, all these genes proved to be DEGs in a well-characterized in vitro model based on a unique cell line and the gene expression kinetics in a complex in vivo system, where the multitude of key players interacting is unknown. Second, the previous in vitro model used represented an initial host–pathogen interaction, as the transcriptome was studied at 12 and 32 hpi. Herein, it is difficult to determine the chronobiology of the disease, but acutely infected males had been infected for at least a few days until 2–3-weeks post-infection, and chronically infected males were infected from 1-month post-infection onwards. Accordingly, it is difficult to extrapolate results obtained in an in vitro model to a complex in vivo system. This asynchrony between studies could explain the absence of significant variations in the gene expressions of genes related with endothelial injury: the loss of endothelial protection (SERPINE), the initial steps of endothelial activation and leukocyte chemotaxis, migration and recruitment to the injury site (VCAM, SELE, CCL2, CXCL2, and ADAMTS1).

Based on the results obtained herein and in a previous study, the inflammation that follows endothelial injury seems to be an initial key pathogenic mechanism responsible for testicular degeneration [11]. One of the main clinical signs that appears in acutely affected males is orchitis [11], and the inflammatory infiltrate of lymphocytes and macrophages is frequently found to be associated with damage of the seminiferous tubules resulting in focal or severe alterations of spermatogenesis [43]. In our study, both immune cells were abundant in the testicular parenchyma from bulls with acute and chronic infection. Infiltrating immune cells are likely to induce a pro-inflammatory microenvironment that might be responsible for the impairment of spermatogenesis in orchitis, as indicated by the pro-inflammatory cytokine Th1 upregulated in the testicular parenchyma of bulls with acute infection. In a later step of the disease subfertility or sterility could be aggravated or maintained by the scrotal skin lesions that impair testicular refrigeration.

This work represents the first approach to identify several molecular and cellular markers that could be indicative of disease progression and sterility. Macrophage infiltrate composed of resident as well as recruited circulating macrophages seems to play a key role in the pathogenesis and could be responsible for the highest gene regulation in the testicular parenchyma of severely affected and sterile bulls. The acute phase is mainly characterized by the upregulation of IL-1α, IL-6 and TIMP1, whereas in the chronic phase, the upregulation of ICAM and the downregulation of MMP13, PLAT and IL-1α were characteristic. All of these genes could be prognosis markers of sterility, as a different gene expression pattern was observed in sub-clinically infected calves. In contrast, a predominance of downregulated genes related with endothelial injury and the pro-inflammatory response and upregulation of CCL24 in the scrotal skin could indicate a subclinical infection. As the routine use of the testicular parenchyma as a tissue sample to monitor disease progression does not seem to be straightforward, further work should be performed in order to identify biomarkers in serum samples.

## Supplementary Information

**Additional file 1: Primers used for RT-PCR expression genes.**

**Additional file 2: Antibodies and procedures used for the immunohistochemical examination.**

**Additional file 3:** **Quantification of the immunohistochemically labelled T and B lymphocytes and phagocytic cells in the testes**. Numbers represent the percentage of cell populations in each tissue analysed. Percentages are representative of three randomly selected medial tissues from each male, and cells were counted in 20 random 20 × fields of each tissue.

**Additional file 4: Transcripts that showed significant variation in the gene expression in testicles from calves.** Non-infected calves [●], infected calves via intravenous [■], subcutaneous [▲] and intradermal [▼]. Data are presented as individual points. Horizontal lines represent median values with the 95% CI for each group. ****, ***, ** and * indicate *P* < 0.0001, *P* < 0.001, *P* < 0.01 and *P* < 0.05 significant differences, respectively.

## Data Availability

The datasets supporting the conclusions of this article are included within the article and its additional files.

## Abbreviations

ACTB: B-Actin
ADAMTS: A disintegrin and metalloproteinase with thrombospondin motif;
CCL: Chemokine (C–C motif) ligand
Ct: Cycle threshold value
CXCL: Chemokine (C-X-C motif) ligand
DEG: differentially expressed genes
dpi: days post-infection
ECM: Extracellular matrix
ELISA: Enzyme-linked immunosorbent assay
GAPDH: Glyceraldehyde-3-phosphate dehydrogenase
hpi: hours post-infection
HE: Haematoxylin and eosin
ICAM: Intercellular adhesion molecule
IFN: Interferon
IgG: Immunoglobulin G
IgM: Immunoglobulin M
IHC: Immunohistochemistry
IL: Interleukin
MMP: Metalloproteinase
PAI-1: Plasminogen activator inhibitor-1
PAS: periodic acid-Schiff
PBS: Phosphate buffer saline
PLAT: Tissue type plasminogen activator
PRRs: Pattern recognition receptors
PTA: Proportional target area
qPCR: Quantitative real‑time polymerase chain reaction
SELE: E-Selectin
SERP: Serpine
TGF: Transforming growth factor
TIMP: Matrix metalloproteinase inhibitor
TLR: Toll‑like receptor
TNF: Tumoral necrosis factor
VCAM: Vascular cell adhesion molecule
WB: Western blot
