# Supplementary material for: Identification of molecular biomarkers associated with disease progression in the testis of bulls infected with Besnoitia besnoiti
Source: Vet Res. 2021 Jul 22;52:106. doi: 10.1186/s13567-021-00974-2 (PMC8296687; doi:10.1186/s13567-021-00974-2)
Supplement: Supplementary file 1 — Additional file 1: Primers used for RT-PCR expression genes. [file 13567_2021_974_MOESM1_ESM.doc]

| **Gene name** | **Gene Symbol** | **Primer sequence (5`-3`)** | **Reference** |
| --- | --- | --- | --- |
| Interleukin 1 alpha | IL1α | Fw: CTCTCTCAATCAGAAGTCCTTCTATG  Rv: CATGTCAAATTTCACTGCCTCCTCC | [14] |
| Interleukin 4 | IL4 | Fw: CTGCCCCAAAGAACACAACT  Rv: GTGCTCGTCTTGGCTTCATT | [16] |
| Interleukin 6 | IL6 | Fw: CTGGGTTCAATCAGGCGATT | [14] |
|  |  | Rv: GGATCTGGATCAGTGTTCTGA |  |
| Interleukin 8 | IL8 | Fw: CCACACCTTTCCACCCCAAA | [16] |
|  |  | Rv: CTTGCTTCTCAGCTCTCTTC |  |
| Interleukin 10 | IL10 | Fw: TGCTGGATGACTTTAAGGGTTACC | [16] |
|  |  | Rv: AAAACTGGATCATTTCCGACAAG |  |
| Interleukin 17 | IL17 | Fw: CACAGCATGTGAGGGTCAAC | [16] |
|  |  | Rv: GGTGGAGCGCTTGTGATAAT |  |
| Tumor Necrosis Factor- | TNF-α | Fw: CCAGAGGGAAGAGCAGTCC | [16] |
|  |  | Rv: GGAGAGTTGATGTCGGCTAC |  |
| Transforming growth factor-beta | TGF-β1 | Fw: GGTGGAATACGGCAACAAAA | [16] |
|  |  | Rv: CGAGAGAGCAACACAGGTTC |  |
| Chemokine (C-X-C motif) ligand 2 | CXCL2 | Fw: TGGTCAGGAAGTGTGTCTCAA | [14] |
|  |  | Rv: TCAGTTGGCACTAGCCTTGTT |  |
| Chemokine (C-C motif) ligand 2 (MCP-1) | CCL2 | Fw: TGCAGACCCCAAGCAGAAAT | [14] |
|  |  | Rv: AGAGGGCAGTTAGGGAAAGC |  |
| Chemokine (C-C motif) ligand 24 | CCL24 | Fw: TAGAGGGCTCTTGGTCACA | [14] |
|  |  | Rv: GTCCTCCAGGTCCATTCATTAC |  |
| Intercellular Adhesion Molecule 1 | ICAM-1 | Fw: AGACCTATGTCCTGCCATCG | [14] |
|  |  | Rv: GGTGCCCTCCTCATTTTCCT |  |
| Vascular cell adhesion molecule 1 | VCAM-1 | Fw: GAACTGGAAGTCTACATCTC | [14] |
|  |  | Rv: CAGAGAATCCGTGGAGCTGG |  |
| E-Selectin | SELE | Fw: CAATACAGGTGTGGTAGGGAC | [14] |
|  |  | Rv: TCATGCCTTGCTAGGG |  |
| Tissue type plasminogen activator | PLAT | Fw: CCACCCTGTTTTCAGCTAAAG | [14] |
|  |  | Rv: TGCTGGGCTCTTGTGACT |  |
| Toll-like receptor 2 | TLR-2 | Fw: ACGACGCCTTTGTGTCCTAC | [16] |
|  |  | Rv: CCGAAAGCACAAAGATGGTT |  |
| A disintegrin and metalloproteinase with thrombospondin motif | ADAMTS1 | Fw: CGGAAAAACCTTTAGAATGGAACA | [14] |
|  |  | Rv: AGGCCCGCTGCCAAA |  |
| Matrix Metallopeptidase 13 (collagenase 3) | MMP13 | Fw: GGAACTAAAGAGCACGGTGAC | [16] |
|  |  | Rv: GGCAGCGACAAGAAACAAG |  |
| Metallopeptidase inhibitor 1 | TIMP1 | Fw: TCGTGGGGACCGCAGAAGT | [16] |
|  |  | Rv: CTCCATGGCAGGGGTGTAGAT |  |
| Stress-associated endoplasmic reticulum protein 1 | SERP-1 | Fw: GACCCTTCACCAAAGATGAG | [16] |
|  |  | Rv: CTGCGAAATTCAGGATGCGGA |  |
| Glyderaldehyde 3-phosphate dehydrogenase | GAPDH | Fw: ATCTCGCTCCTGGAAGATG | [14] |
|  |  | Rv: TCGGAGTGAACGGATTCG |  |
| B-Actin | ACTB | Fw: ACACCGCAACCAGTTCGCCAT | [14] |
|  |  | Rv: GTCAGGATGCCTCTCTTGCT |  |
