# Supplementary material for: Identification of molecular biomarkers associated with disease progression in the testis of bulls infected with Besnoitia besnoiti
Source: Vet Res. 2021 Jul 22;52:106. doi: 10.1186/s13567-021-00974-2 (PMC8296687; doi:10.1186/s13567-021-00974-2)
Supplement: Supplementary file 2 — Additional file 2: Antibodies and procedures used for the immunohistochemical examination. [file 13567_2021_974_MOESM2_ESM.docx]

|  | | | | | | | |  |
| --- | --- | --- | --- | --- | --- | --- | --- | --- |
| **Target** | **Specificity of primary antibodies** | **Monoclonal/**  **Polyclonal primary antibody** | **Deparaffinization and epitope retrieval** | **Primary antibody dilution** | **Secondary**  **antibody** | **Reference** |  | |
| CD3 | T cells, thymocytes and natural killer cells | Polyclonal | Citrate-based solution ph 6.0 (PT-Link System®) | 1:300 | Envision+ System-HRP. Rabbit (DAB+) (Dako®) | A0452, Agilent Technologies® |  | |
| CD21 | B-lymphocytes | Monoclonal | Citrate-based solution ph 6.0 (PT-Link System®) | 1:250 | Envision+ System-HRP. Mouse (DAB+) (Dako®) | MCA1424GA, Biorad® |  | |
| Iba-1 | Microglia/macrophage | Polyclonal | Tris-based solution pH 9.0 (PT-Link System®) | 1:1000 | Envision+ System-HRP. Rabbit (DAB+) (Dako®) | NCNP24, 019-19741, Wako® |  | |
| MAC387 | Recently recruited tissue macrophages | Monoclonal | Tris-based solution pH 9.0 (PT-Link System®) | 1:200 | Envision+ System-HRP. Mouse (DAB+)(Dako®) | GTX39774, Genetex® |  | |
