# Supplementary material for: Identification of molecular biomarkers associated with disease progression in the testis of bulls infected with Besnoitia besnoiti
Source: Vet Res. 2021 Jul 22;52:106. doi: 10.1186/s13567-021-00974-2 (PMC8296687; doi:10.1186/s13567-021-00974-2)
Supplement: Supplementary file 3 — Additional file 3: Quantification of the immunohistochemically labelled T and B lymphocytes and phagocytic cells in the testes. Numbers represent the percentage of cell populations in each tissue analysed. Percentages are representative of three randomly selected medial tissues from each male, and cells were counted in 20 random 20 × fields of each tissue. [file 13567_2021_974_MOESM3_ESM.docx]

| Group | Bulls | CD3 | | |  | | CD21 | | |  | | Iba-1 | | | |  | | | MAC387 | | |  |
| --- | --- | --- | --- | --- | --- | --- | --- | --- | --- | --- | --- | --- | --- | --- | --- | --- | --- | --- | --- | --- | --- | --- |
|  |  | Scrotal skin | Pampiniform plexus | Testicular parenchyma | |  | Scrotal skin | Pampiniform plexus | Testicular parenchyma | |  | | Scrotal skin | Pampiniform plexus | Testicular parenchyma | |  | Scrotal skin | | Pampiniform plexus | Testicular parenchyma | |
| Non-infected bulls | No. 3 | 5.27 | 3.36 | 4.70 | |  | 0.00 | 0.00 | 0.00 | |  | | 7.27 | 4.68 | 10.1 | |  | 0.00 | | 0.00 | 7.88 | |
|  | No. 5 | 9.23 | 0.00 | 4.30 | |  | 0.72 | 0.00 | 0.00 | |  | | 7.52 | 2.21 | 4.76 | |  | 2.71 | | 0.00 | 0.00 | |
| Acutely infected bulls | No. 3 | 7.01 | 0.00 | 2.44 | |  | 0.00 | 0.00 | 0.00 | |  | | 14.48 | 0.00 | 11.34 | |  | 0.00 | | 0.00 | 0.00 | |
|  | No. 6 | 7.92 | 7.29 | 6.76 | |  | 0.00 | 0.00 | 0.00 | |  | | 10.79 | 18.90 | 17.01 | |  | 1.87 | | 1.61 | 4.34 | |
| Chronically infected bulls | No. 9 | 3.68 | 0.00 | 3.70 | |  | 0.00 | 0.00 | 0.00 | |  | | 0.63 | 0.70 | 5.80 | |  | 0.00 | | 0.00 | 0.00 | |
|  | No. 10 | 4.60 | 0.00 | 0.00 | |  | 0.00 | 0.00 | 0.00 | |  | | 3.70 | 0.96 | 8.50 | |  | 0.00 | | 0.00 | 0.00 | |
